# Nanoparticle Delivery of CRISPR/Cas9 for Genome Editing

**DOI:** 10.3389/fgene.2021.673286

**Published:** 2021-05-12

**Authors:** Li Duan, Kan Ouyang, Xiao Xu, Limei Xu, Caining Wen, Xiaoying Zhou, Zhuan Qin, Zhiyi Xu, Wei Sun, Yujie Liang

**Affiliations:** ^1^Department of Orthopedics, The First Affiliated Hospital of Shenzhen University, Shenzhen Second People’s Hospital, Shenzhen, China; ^2^Shenzhen Institute of Geriatrics, Shenzhen, China; ^3^Guangdong Provincial Research Center for Artificial Intelligence and Digital Orthopedic Technology, Shenzhen Second People’s Hospital, Shenzhen, China; ^4^Department of Child and Adolescent Psychiatry, Shenzhen Kangning Hospital, Shenzhen Mental Health Center, Shenzhen Key Laboratory for Psychological Healthcare & Shenzhen Institute of Mental Health, Shenzhen, China

**Keywords:** nanocarriers, CRISPR/Cas9, delivery, exosome, modification

## Abstract

The emerging clustered regularly interspaced short palindromic repeat (CRISPR)/CRISPR-associated system (Cas) gene-editing system represents a promising tool for genome manipulation. However, its low intracellular delivery efficiency severely compromises its use and potency for clinical applications. Nanocarriers, such as liposomes, polymers, and inorganic nanoparticles, have shown great potential for gene delivery. The remarkable development of nanoparticles as non-viral carriers for the delivery of the CRISPR/Cas9 system has shown great promise for therapeutic applications. In this review, we briefly summarize the delivery components of the CRISPR/Cas9 system and report on the progress of nano-system development for CRISPR/Cas9 delivery. We also compare the advantages of various nano-delivery systems and their applications to deliver CRISPR/Cas9 for disease treatment. Nano-delivery systems can be modified to fulfill the tasks of targeting cells or tissues. We primarily emphasize the novel exosome-based CRISPR/Cas9 delivery system. Overall, we review the challenges, development trends, and application prospects of nanoparticle-based technology for CRISPR/Cas9 delivery.

## Introduction

Gene-editing technology is sometimes called “God’s scalpel,” which can precisely and efficiently prune, cut, replace, or insert DNA or RNA sequences. Since its appearance in the 1980s, gene-editing technology has made continuous breakthroughs and quickly moved toward medical applications. The development of gene-editing technologies, such as zinc finger nuclease (ZNF), transcription activator-like effector nuclease (TALEN), and clustered regularly interspaced short palindromic repeat (CRISPR), belonging to the third generation of gene-editing technology, has guided life science research into a new era (Gaj et al., 2013; Gupta et al., 2019).

CRISPR/Cas9 gene-editing technology won the 2020 Nobel Prize in Chemistry. CRISPR has become the most effective and convenient tool for gene editing and has widespread applications in biomedical research and clinical investigation. Although CRISPR technology’s application to edit DNA has made outstanding achievements in recent years, it still needs improvement.

As the CRISPR/Cas9 complex needs to operate on the nuclear genome, its components need to be transferred into the nucleus. Therefore, it is necessary to overcome the barriers of tissues and the cell membrane. Efficient delivery of CRISPR/Cas9 to target tissues or cells faces considerable challenges. The current CRISPR/Cas9 delivery methods include non-viral vectors, viral vectors, and physical delivery. Virus-mediated gene delivery is the most widely used method and it involves integrating CRISPR/Cas9-encoding sequences into the viral genome and releasing the CRISPR/Cas9 gene complex into infected cells. During this process, the viral vectors may integrate into host cells and cause problems such as mutations, carcinogenesis, and an immune response (Xu et al., 2019; Yip, 2020).

Additionally, the virus is limited to delivering CRISPR/Cas9 DNA, and its load capacity is minimal (Chew et al., 2016). For example, adeno-associated viruses can only load up to 4.7 kb DNA sequences (Zincarelli et al., 2008). Therefore, their ability to deliver the CRISPR/Cas9 system safely and efficiently remains to be determined. Electroporation is the most common physical method to transfer CRISPR/Cas systems into cells. Single-cell microinjection, another physical delivery tool of CRISPR/Cas9, has been widely used in embryo gene editing and transgenic animal production. The transfer of Cas9 DNA or protein components has shown high transduction efficiency and low cytotoxicity. However, microinjection is time-consuming and labor intensive, which limits its application to a small number of species.

Recently, nanomaterials have gradually shown unique advantages in gene delivery. Several nano-delivery systems for CRISPR/Cas9 have been developed, including cationic liposomes, lipid nanoparticles (LNPs), cationic polymers, vesicles, and gold nanoparticles (Chin et al., 2019; Chen et al., 2020). As the most promising non-viral nanocarrier, exosomes can efficiently deliver the CRISPR/Cas9 system *in vitro* and *in vivo*. They serve as unique tools to expand the use of this powerful gene-editing technology in the life sciences and in clinical applications. Thus, in this review, we briefly summarize the current CRISPR components (Cas9 and sgRNA) involved in nano-delivery. We focused on developing new nano-delivery vector tools and their applications of CRISPR/Cas9, paying particular attention to the latest exosome-based technology for CRISPR/Cas9 delivery.

## Delivery Cargoes of the CRISPR/Cas9 System

**Figure 1:**
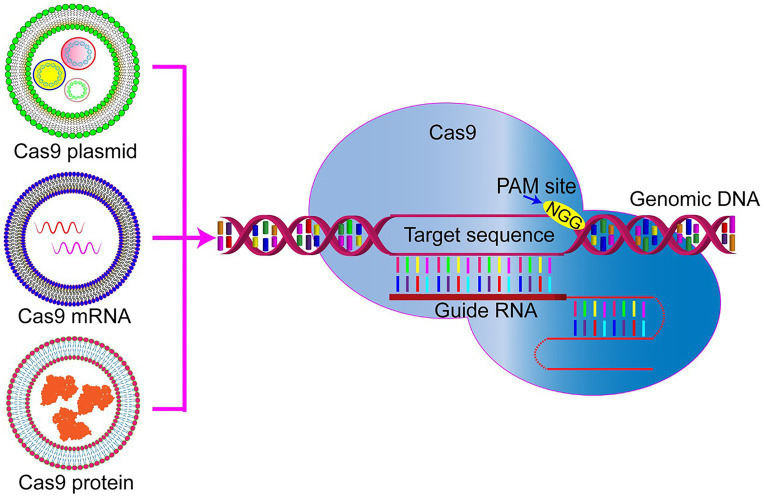
Representative genome editing by three forms of clustered regularly interspaced short palindromic repeat (CRISPR)/Cas9. Cas9 plasmid DNA, RNA, or protein delivery *via* nanoparticles to be used for precise genome editing. PAM, protospacer adjacent motif.

The CRISPR/Cas9 gene-editing system is unique because it requires co-delivery of two components, namely, Cas9 plus single guide RNA(s). At present, three types of cargoes are delivered (Figure 1). One of the delivered cargoes is a protein, namely the Cas9 endonuclease protein complexed with sgRNA to form a ribonucleoprotein (RNP; Woo et al., 2015; Zuris et al., 2015). Delivering an RNP is the most straightforward strategy, with no need for a transcription or translation process. It quickly starts genome editing when entering the cell, significantly reducing off-target effects and immune responses. However, this strategy requires the purification of highly active Cas9 protein. Additionally, it is challenging for it to enter cells due to the high molecular weight of the Cas9 protein. Therefore, developing nanodrug delivery systems that can carry RNP cargoes is essential. At present, protein delivery is the priority option only in embryonic microinjection (Chang et al., 2013; Chaverra-Rodriguez et al., 2018).

Compared with protein delivery strategies, the transfection of plasmids encoding CRISPR/Cas9 or sgRNA is much easier to perform by extracting the plasmid and then loading it into the nanocarrier. Knocking-in or gene mutation is performed by integrating the corresponding plasmids into the same delivery plasmid. However, the gene fragment size encoding the CRISPR/Cas9 system is often too large, leading to a low transfection efficiency.

Another possibility for delivered cargo is mRNA. Cas9 mRNA and sgRNA can be prepared by *in vitro* transcription, transferred into the cells, and directly translated into protein in the cytoplasm to exert their genome editing function. The relatively low stability of mRNA and its subsequently limited expression time are the main obstacles hindering its application. It is challenging to deliver mRNA *in vivo* for disease treatment. So far, this strategy has only been used to edit the genomes of embryos, zygotes, embryos, and cultured cells (Shen et al., 2014; Miller et al., 2017).

However, regardless of the delivery cargo, it is challenging for the CRISPR/Cas9 to enter cells. Due to its considerable molecular weight (the genetic size of Cas9 ~4.5 kb) and its poor stability, finding a more suitable nano-delivery method for the various Cas9 components is vital. When designing and preparing a delivery system, it is necessary to focus on maintaining the nuclease activity of Cas9 and protecting the RNP against proteases, nucleases, antibodies, and T cell recognition in the serum and body fluids. Once entering the target cell, the delivery system should help the RNP be released from the endosome to the cytoplasm and enable its function.

**Figure 2:**
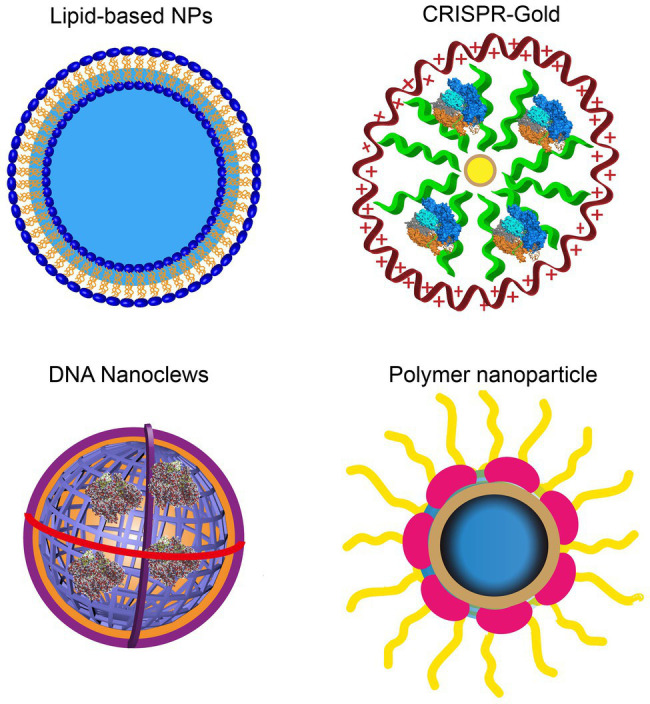
Rational designs of nano-delivery vehicles for plasmid-, RNA-, and RNP-based CRISPR/Cas9. DNA nanostructures show the controllable size and architecture assembly. Gold nanoparticles show high delivery efficiency.

Currently, nanocarriers are ideal delivery platforms for the CRISPR/Cas9 system, including cationic LNPs, DNA nanoparticles, lipid complexes, gold-based nanoparticles, and zeolite imidazole frameworks. They have been used for *in vitro* RNP delivery under extensive development and application efforts (Figure 2; Yin et al., 2016). Table 1 lists the current nanoplatforms for CRISPR/Cas9 delivery.

**Table 1:** Nanoparticle delivery of CRISPR/Cas9.

Delivery approaches	NP formulation	Characterization	CRISPR/Cas9 cargo	Efficiency	Application	References
Lipid nanoparticle	Bioreducible lipid-like materials: cholesterol, 1,2-dioleoyl-*sn*-glycero-3-phosphoethanolamine (DOPE), and C16-PEG_2000_-ceramide	Bioreducible	Cas9: sgRNA complexed	70% (HEK293-GFP)	*in vitro* and *in vivo*	Wang et al., 2019
	Amino-ester-derived lipid like nanomaterials	Biodegradability and low toxicity	Cas9 mRNA and sgRNA	41% (eGFP signal)	*in vitro* and *in vivo*	Zhang et al., 2017
	Lipid-like nanomaterials: FTT lipids, 1,2-dioleoyl-sn-glycero-3-phosphoethanolamine (DOPE), cholesterol, and 1,2-dimyristoyl-rac-glycero-3-methoxypolyethylene glycol-2000 (DMG-PEG_2000_)	High delivery efficiency and biodegradability	Cas9 mRNA and sgRNA	~60% (*in vivo* base editing of PCSK9)	*in vivo*	Shi et al., 2020
	Lipid nanoparticle: ionizable lipid (LP01), cholesterol, and DSPC		Cas9 mRNA and sgRNA	>97% [transthyretin (*Ttr*) in serum]	*in vitro* and *in vivo*	Finn et al., 2018
	Zwitterionic amino lipids: cholesterol:PEG-lipid	Permanent DNA editing	Cas9 mRNA and sgRNA	95% (HeLa-Luc-Cas9) 1.5–3.5% (Hepatocytes)	*in vitro* and *in vivo*	Miller et al., 2017
Polymer	Chitosan	High efficacy and non-cytotoxicity	Cas9 RNPs	12.50%	*in vitro*	Qiao et al., 2019
	Carboxymethyl chitosan with AS1411 ligands	Dual-targeting delivery and high efficiency	pDNA	>90% (CDK11 protein)	*in vitro*	Liu et al., 2018
DNA nano-structure	DNA nanoclews	Stable assembly and multiple editing	RNP	80% (EGFP in U2OS)	*in vitro*	Sun et al., 2015b
	Branched DNA-based nanoplatform	Biocompatible	RNP	40% (genetic cleavage)	*in vitro* and *in vivo*	Liu et al., 2019
	MicroRNA-responsive DNA nanoclews	Stimuli-responsive release	RNP	45% (EGFP in HeLa)	*in vitro* and *in vivo*	Shi et al., 2020
Inorganic nanomaterials	Cationic arginine gold nanoparticles (ArgNPs)	High delivery efficiency	RNP	∼90% (delivery efficiency) 23–30% (gene editing efficiency)	*in vitro*	Yang et al., 2011; Mout et al., 2017; Mout and Rotello, 2017
	CRISPR-Gold	Biocompatibility	RNP	40–50% (mGluR5 protein and mRNA)	*in vitro* and *in vivo*	Lee et al., 2018
	CRISPR-Gold	Low local immunogenicity, multiple uses	RNP	5.40% (restoration of Duchenne muscular dystrophy)	*in vivo*	Lee et al., 2017
Exosome	Exosome-liposome hybrid	Efficiently encapsulate plasmid	pDNA	∼40% (Runx2 mRNA)	*in vivo*	Lin et al., 2018
	VSV-G protein ectosomes: split GFP	Efficient delivery	Cas9 protein	_	*in vitro* and *in vivo*	Zhang et al., 2020
	Engineered exosome: GFP-GFP nanobody	Efficient delivery	Cas9 protein	_	*in vitro*	Ye et al., 2020
	Engineered exosome: CD9-HuR exosomes	Enhanced encapsulation	Cas9 mRNA	_	*in vitro* and *in vivo*	Li et al., 2019
	NanoMEDIC	Efficient delivery and high cleavage activity	Cas9 protein	90% (exon skipping efficiencies)	*in vitro* and *in vivo*	Gee et al., 2020
	Red blood cell (RBC)-derived EVs	Efficient loading and delivery	Cas9 mRNA	~32% (gene silencing) ~18% (loading capacity Cas9 mRNA)	*in vitro* and *in vivo*	Usman et al., 2018

## CRISPR/Cas9 Nano-Delivery Approaches

### Lipid-Based Nanoparticles

Lipid nanoparticles, the classic delivery systems for nucleic acid transfer, have been extensively studied. Nucleic acids with negative charges and lipids with positive charges form a complex by host-guest interactions and electrostatic interactions and are then taken up by endocytosis. Nanoparticles deliver plasmids or mRNAs and can protect them from nuclease degradation. LNPs can transport small interfering RNA (siRNA) and mRNA, which has been extensively validated in preclinical experiments and clinical studies. However, the editing efficiency of using LNPs to package CRISPR/Cas9 plasmids has not met the clinical requirements because their delivery efficiency to primary cells or *in vivo* animal experiments is low. Modification of the lipid nanodrug delivery system could significantly improve its delivery efficiency.

The difficulties in controlling their size, uniformity, and stability severely limit local administration of LNPs *in vivo*, such as to the inner ear, muscle, brain, and tumors. At present, developing stable nanoparticles for systemic delivery of RNPs to target organs is still challenging. Researchers have developed a universal engineering method to maintain the integrity of RNP by adding permanent cationic supplements (such as DOTAP) to ionizable LNP formulations. The lipid components mediate the encapsulation of C RNPs with retained activity and redirect DNA editing to the target tissues, so that low-dose intravenous injections can effectively target specific tissues, including the sphincter muscles, brain, liver, and lungs (Wei et al., 2020).

Biodegradable lipid-like nanoparticles (LLNs) are functionalized liposomes that effectively deliver CRISPR/Cas9 cargoes and reduce liposome biological toxicity. Zhang et al. (2017) designed and synthesized a series of biodegradable lipid-like compounds containing ester groups to deliver Cas9 mRNA *in vitro* and *in vivo*. Studies have also demonstrated that biodegradable lipids in the intracellular environment can improve liposome escape and cargo release. Therefore, integrating reducible disulfide bonds into the hydrophobic tail of lipids can improve their gene delivery efficiency and promote endosomal escape.

Lipid nanoparticles can be formulated with biodegradable ionizable lipids, PEG-DMG, Spy Cas9 mRNA, and sgRNA. They can effectively deliver CRISPR/Cas9 components *in vivo* for genomic editing and produce sustainable gene knockout within 52 weeks after a single administration. The LNP formulation decreased mouse TTR protein expression >97%, indicating the therapeutic role of CRISPR/Cas9-based gene editing in the liver (Finn et al., 2018).

These lipid-like nanomaterials are worthy of further development as delivery tools for gene therapy. Miller et al. (2017) synthesized zwitterion amino lipids, which can deliver long RNAs, including Cas9 mRNA and targeted sgRNA, for a sustained 95% protein reduction. This is the first non-viral delivery system reported for co-delivery of Cas9 mRNA and sgRNA *in vitro* and *in vivo*.

Guo et al. (2019) developed an antibody-conjugated tumor-targeted nanolipogel that can precisely deliver CRISPR/Cas9 plasmids into triple-negative breast cancer. Eighty-one percent knockout efficiency of the Lipocalin 2 gene led to a tumor growth rate reduction of 77%. These studies demonstrated that tNLGs serve as a safe, accurate, and effective delivery platform for target-specific precision of CRISPR-mediated genome editing.

Altogether, in addition to easy preparation, LNPs are safe and suitable for CRISPR/Cas9 DNA and mRNA delivery. Although commercial Lipofectamine cannot meet CRISPR/Cas9 delivery requirements, functional lipid modifications can provide a new generation of high-efficiency delivery systems for gene editing.

### Polymer-Based Nanoparticles

Compared with cationic lipid carriers, cationic polymer carriers show chemical diversity and functional potential, providing more choices for flexible structural designs. Cationic polymer nanoparticles have been widely used to deliver different nucleic acid types, including plasmid DNA and mRNA. Cationic polymers, such as polyethyleneimine and chitosan, were the most frequently used carriers for CRISPR/Cas9 delivery. Similar to lipid carriers, polymer nanoparticle carriers can traverse the membrane through endocytosis and protect the loaded cargoes from the immune response and nuclease degradation.

Recently, studies have focused on improving CRISPR/Cas9 delivery efficiency *in vivo*, thereby optimizing disease treatment *via* gene editing. Yin et al. (2013) prepared the α-helical cationic polyamine acid PPABLG, showing its ability to penetrate the cell membrane and its high-efficiency endosomal escape. The plasmids encoding Cas9/sgRNA mixed with copolymers could achieve up to 60% Cas9 expression and knockout up to 35% of the polo-like kinase gene. The targeted and environment-responsive delivery of CRISPR/Cas9 significantly reduced off-target effects.

Recently, the amphiphilic block polymer polyethylene glycol-b-poly (lactic-glycolic acid; PEG-b-PLGA), with the assistance of cationic lipids, was used to load Cas9 mRNA or CRISPR/Cas9 plasmids for intracellular delivery to macrophages (Luo et al., 2018). Efficient specific gene editing in macrophages can be achieved with an intravenous injection of a macrophage-specific promoter driving Cas9 expression. A multifunctional nuclear-targeted nuclear-shell structure has been developed using perfluorobutanamide-modified oligoPEI and polypeptide RGD-R8-modified hyaluronic acid. This system can efficiently load CRISPR/Cas9 plasmids and achieve endosomal escape and nuclear delivery, leading to significant knockout of the target gene.

To make a responsive polymer, researchers have constructed a multistage delivery polymer nanocarrier (MDNP) that responds to the tumor’s slightly acidic environment, thus achieving tumor-targeted delivery of the CRISPR/Cas9 system. By designing the core-shell structure MDNP, the shell made of a responsive polymer can respond to the tumor microenvironment, so that MNDP can overcome various physiological barriers and achieve targeted delivery of CRISPR/Cas9 to inhibit tumor growth (Liu et al., 2019).

Additionally, chitosan is a convenient polymer carrier for CRISPR/Cas9 delivery. Qiao et al. (2019) encapsulated red fluorescent protein (RFP) in chitosan to form positively charged NPs, simultaneously delivering Cas9 RNP along with 20 glutamate residues. The ssDNA donor is simultaneously delivered to the cytoplasm and then released and transferred to the nucleus for HDR-mediated genome editing. Additionally, Liu et al. (2018) designed dual-targeted polymer/inorganic hybrid nanoparticles. In this system, with co-precipitation, the cyclin-Dependent Kinase 11 (CDK11) knockout CRISPR/Cas9 plasmid is encapsulated in the core of the nano-delivery system made up of protamine sulfate, calcium carbonate, calcium phosphate, and carboxymethyl chitosan. The S1411 aptamer ligands can interact with each other through an electrostatic effect. This dual-targeted polymer nano-system can deliver the CRISPR/Cas9 plasmid to the nucleus of tumor cells and efficiently knockout genome CDK11. Through a flexible structural design, polymeric vectors could be formulated with the CRISPR/Cas9 system and achieve ideal therapeutic effects.

### DNA Nanostructures

DNA has been used to construct DNA nanostructures for imaging and targeted drug delivery. The DNA sequence is controllable, and the interactions of DNA are predictable, thus allowing DNA to self-assemble into complex DNA nanostructures. DNA nanostructures are an emerging delivery system with the advantages of a strong loading capacity, biocompatibility, and biodegradability. Traditionally, DNA nanostructures are mediated by Watson-Crick base pairing of short DNA fragments. This assembly process is complicated and requires a large amount of DNA. Recently, rolling circle amplification (RCA) has simplified the DNA assembly of nanostructures (Sun et al., 2015b). Sun et al. (2015a) developed a new type of self-assembled yarn-like DNA nanomolecular through rolling circle amplification, which can be used to deliver CRISPR/Cas9 RNP *in vitro* and *in vivo*.

DNA nanostructures can be modified to target specific membrane receptors, enabling receptor-dependent clathrin‐ or caveolae-mediated pathways for effective cellular uptake. Chemical modifications can further improve the physiological stability of DNA nanostructures. In one study, when the Cas9/sgRNA/DNA nanomolecular complex surface was coated with a cationic PEI polymer, cellular uptake and endosomal escape were significantly enhanced, and the genome editing efficiency was approximately 28% (Sun et al., 2015a).

DNA nanomaterials can also be used to encode DNA aptamers to target specific tumor cells and deliver a miRNA response system. Researchers have demonstrated that using nanoflowers combined with a stimulus-responsive Cas9/sgRNA release strategy can significantly enhance genome editing efficiency. The release of miR-21-responsive Cas9/sgRNA can achieve cell type-specific targeting (Shi et al., 2020).

### Gold Nanoparticles

Gold nanoparticles are a new type of CRISPR/Cas9 RNP delivery vehicle. It is easy to cross-link gold nanoparticles (AuNPs) with sulfhydryl (-SH) substances through Au-S bonds and manipulate their surface charge and hydrophilicity (Levy et al., 2010). After constructing AuNP surface-modified cationic peptides, pCas9 can be adsorbed by electrostatic interactions. Wang et al. (2018) modified the TAT peptide (C-terminal cysteine) on the surface of AuNPs carrying the pCas9 protein. After intravenous administration, Cas9 is released by a thermal effect triggered by a laser directed to the AuNPs. The cationic TAT peptide can guide pCas9/sgPLK-1 (Polo-like kinase 1) to enter the nucleus and destroy the PLK-1 gene, thus inhibiting tumor growth. This AuNP concentrated, lipid-encapsulated, and laser-controlled delivery system has efficient CRISPR/Cas9 delivery and targeted gene editing. Researchers may use it to treat various diseases.

Mout et al. (2017) designed cationic arginine-functionalized gold nanoparticles (ArgNPs) to deliver chemically modified Cas9 protein and sgRNA. Cas9 was modified with a glutamate peptide tag at the N-terminus. This negatively charged amino acid peptide tag can neutralize the positive charge of the Cas9 protein, bind to the positively charged arginine residues on ArgNPs, and then form self-assembled nanocomponents. This Cas9 protein delivery strategy showed high efficiency cytoplasmic/nuclear delivery (~90%), and its genome editing efficiency was between 23 and 30% (Yang et al., 2011; Mout et al., 2017; Mout and Rotello, 2017). The AuNP core was coupled with SH-spacer monomers of oligo ethylene glycol (OEG)-crRNA for the loading of Cas9 onto the nanoparticle by its natural affinity with crRNA. Then, the complex was packaged with PEI, and the donor single-stranded DNA (ssDNA) was adsorbed on the surface through electrostatic interactions. They demonstrated that RNP-loaded AuNPs could efficiently deliver Cas9-targeted HDR to hematopoietic stem and progenitor cells (HSPCs) *in vitro* (Shahbazi et al., 2019).

More interestingly, the development of CRISPR-Gold technology accelerated the applications of AuNPs in the field of gene editing. CRISPR-Gold can regulate the amount necessary per injection and reduce the CRISPR technology’s side effects, such as off-target effects. By using AuNP surfaces coupled with thiol-modified oligonucleotides (DNA-thiol), DNA-thiol can hybridize with donor ssDNA, and the Cas9 RNP is loaded by the affinity of ssDNA and Cas9 nuclease. Subsequent cationic polymer PAsp (DET) encapsulation generates CRISPR-Gold. CRISPR-Gold can repair the mutant dystrophin gene after intramuscular injection with a correction rate of 5.4% and reduce muscle fibrosis in X-linked muscular dystrophy (MDX) mice (Lee et al., 2017). As CRISPR-Gold technology can precisely edit specific brain cells, it suggests a possibility for the future application of CRISPR technology in various neurogenetic diseases and targeted treatment of social disorders. Intracranial delivery of RNA-guided endonucleases Cas9 and Cpf1 to adult mouse brains by CRISPR-Gold and targeted knockout of the metabotropic glutamate receptor 5 (mGluR5) gene can effectively reduce mGluR5 levels and reverse the repetitive behaviors of autism caused by fragile X syndrome (Lee et al., 2018). CRISPR-Gold technology is not limited to the treatment of single-gene diseases but can also be expanded to treat polygenic diseases such as Huntington’s disease by dual sgRNA. Due to the excellent fluorescence emission and adjustable surface functionalization of gold nanoparticles, AuNCs can be used for real-time monitoring of biological effects while editing genes (Tao et al., 2021). Developing more AuNC-based nanocomposites will enable broad applications of CRISPR in diagnostics and therapy.

### Extracellular Vesicles-Based Delivery of Cas9/gRNA

The greatest challenge in the clinical application of the CRISPR/Cas9 system is the lack of a safe and effective delivery system. Exosomes are natural membrane-bound vesicles exhibiting the advantages of high biocompatibility and low immunogenicity. They can carry various biomolecules, including plasmids, proteins, siRNAs, and miRNAs, that could serve as cell-free therapies (Duan et al., 2020b; Liang et al., 2021b). Thus, engineered exosomes have been used for targeted drug delivery (Duan et al., 2021; Liang et al., 2021a). Our studies have already proven the potential of targeted exosomes for miRNA and small molecular delivery in cartilage repair (Duan et al., 2020a; Liang et al., 2020; Xu et al., 2021). The functional components of gRNA and Cas9 protein can be encapsulated in exosomes and they can transmit the gene-editing activity of the CRISPR/Cas9 system intracellularly. Exosomes carrying functional gRNA and Cas9 proteins can be produced using bioengineered Vero and CHO cells. Exosomes can also deliver gRNA and Cas9 proteins to the target cells and achieve functional delivery of the CRISPR/Cas9 system (Chen et al., 2019). Due to their excellent biocompatibility and the rapid degradation of gRNA and Cas9 proteins inside cells, the delivery of gRNA and Cas9 reduces the risks of off-target effects and integrating into the genome. Therefore, exosomes may be the safest and most effective delivery method for the CRISPR/Cas9 system.

#### Endogenous Exosomes Deliver CRISPR/Cas9 for Tumor Therapy

Exosomes derived from tumor cells can efficiently target and deliver Cas9/sgRNA to tumor tissues with maternal cell tropism. After intravenous injection of exosomes from SKOV3 human ovarian cancer cells (SKOV3-Exos) loaded with CRISPR/Cas9, they target and accumulate in SKOV3 tumors, inhibiting PARP-1 gene expression and subsequently inducing apoptosis of ovarian cancer cells (Kim et al., 2017). CRISPR/Cas9-mediated genome editing can also exhibit synergistic cytotoxicity by enhancing cancer cell drug sensitivity to cisplatin. Therefore, tumor-derived exosomes are very promising for future cancer gene editing. Chen et al. transfected HPV‐ or HBV-specific CRISPR/Cas9 expression plasmids into HeLa cells and HuH7 cells, respectively, and found that gRNA and Cas9 proteins can be encapsulated in endogenous exosomes. In addition, the functional gRNA and Cas9 proteins carried by this exosomal system are transmitted intracellularly, destroying the HBV genome of neighboring cells. Although endogenous exosomes may be used as a safe and effective delivery vector for the CRISPR/Cas9 system, they may complicate the off-target effects and safety issues of gene-editing technology.

#### Hybrid Exosomes Deliver CRISPR/Cas9 Plasmids

At present, exosomes are mainly used as a delivery platform for small nucleic acids such as miRNA and siRNA or small molecular compounds. Due to the small size of exosomes, it is challenging to pack larger fragments of DNA nucleic acid into exosomes. The two separate lipid membranes of exosome and liposome will merge into a single continuous bilayer, which is mediated by lipid physics or by protein-lipid interactions. Incubating the exosomes with liposomes at 37°C for 12 h or using the freeze-thaw method can induce exosomal-liposome membrane fusion.

**Figure 3:**
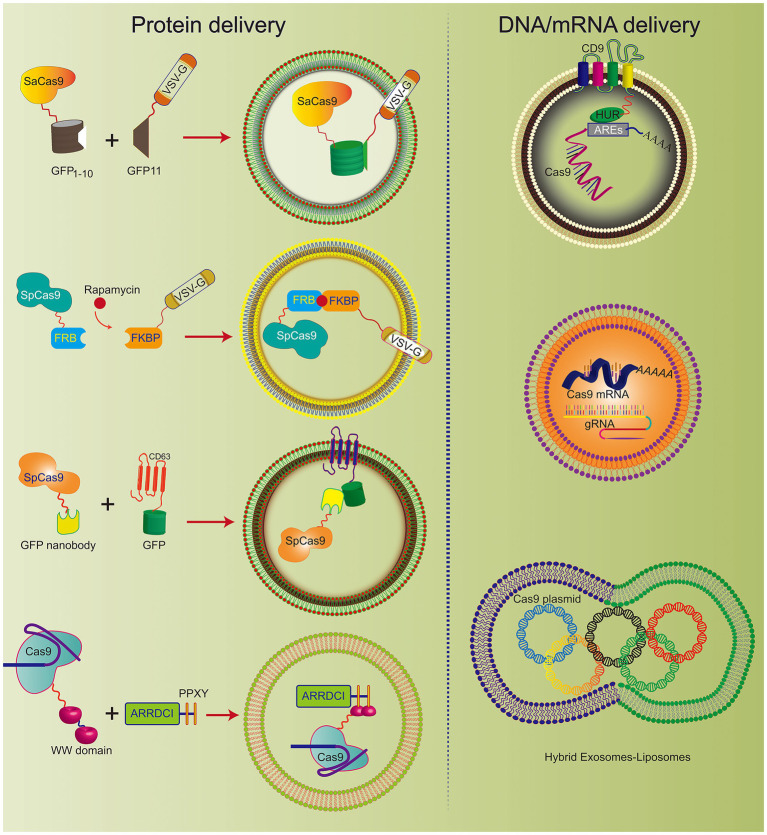
Various engineered exosomes for the efficient delivery of CRISPR/Cas9 components. The co-expression system of SaCas9-GFP1-10 and VSVG-GFP11 can be actively loaded into exosomes. Rapamycin induces an interaction between FK506 binding protein (FKBP)-rapamycin binding domain (FRB) and FKBP, which incorporates the Cas9 complex into the engineered exosome. CD63, as the exosomal member, is fused with GFP and Cas9 protein-linked nanobodies. Thus, the Cas9 protein could be encapsulated into exosomes specifically by the GFP-binding nanobody. CD9-HuR exosomes can actively load AU-enriched mRNA for Cas9 mRNA delivery. Hybrid exosomes with liposomes can effectively load the Cas9 plasmid.

It has been reported that hybrid nanoparticles can significantly increase the loading efficiency of plasmid DNA. The hybrid exosomes efficiently encapsulate large CRISPR/Cas9 expression plasmids (Figure 3). Moreover, hybrid exosomes can be used to transfect mesenchymal stem cells that are not easy to transfect (Lin et al., 2018).

Therefore, exosomes hybridizing with other nanomaterials can overcome several existing limitations of exosomes and improve the application prospects of exosomes as general drug carriers.

#### Engineering Exosomes Deliver Cas9 Protein

CRISPR/Cas9 expression plasmids usually arouse concerns that the integration of DNA fragments may follow genome editing. To improve the safety of CRISPR/Cas9 technology, researchers developed engineered exosomes to load Cas9/gRNA components. The Cas9 protein can be effectively fused into exosomes through the interaction between the inner exosomal membrane protein and the Cas9 fusion protein (Figure 3). For example, Cas9 protein is loaded into exosomes through the interaction between the WW domain and exosome-related protein (ARRDC1). Since the G protein of vesicular stomatitis virus (VSV-G) can be incorporated into exosome-like vesicles (ELVs), it was used to develop active cargo loading by genetic engineering. Based on the auto-assembly of GFP between two polypeptides, GFP1-10 and GFP11 (residues 11 β chains), the Cas9 protein was fused with GFP1-10 and GFP11 by VSVG. Subsequently, Cas9 will be automatically assembled into exosomes by split GFP complementation (Zhang et al., 2020).

The same method was also used for chemically induced Cas9 protein loading. Since rapamycin can induce a dimerization interaction between the FK506 binding protein (FKBP12) and the FKBP-rapamycin binding domain (FRB), the Cas9 protein can be selectively packaged into EVs when Cas9 fuses to FRB and VSVG fuses to FKBP12. This NanoMEDIC effectively induces genome editing of various human cell types, especially pluripotent stem cell (iPSC) gene editing. NanoMEDIC delivers Cas9 with high efficiency to target the scissor acceptor and donor site of iPSCs derived from patients with Duchenne muscular dystrophy (DMD). It can achieve an exon-skipping efficiency of over 90%. The mouse model showed permanent genomic exon skipping, indicating that NanoMEDIC can be used for *in vivo* genome editing treatment of DMD.

In addition, the use of a GFP binding protein (a GFP nanobody) can enrich for sgRNA and Cas9 proteins in exosomes. Two plasmids encoding Cas9-fused GFP nanobodies and CD63-GFP were co-transfected. Since CD63 is an exosomal tetraspan transmembrane protein, GFP fused with the C-terminus of the CD63 protein can be expressed on the inner surface membrane of exosomes. When the Cas9 protein is fused with the GFP-binding nanobody, they can effectively load Cas9 into exosomes.

Campbell et al. (2019) prepared vesicles by co-transfecting vesicular stomatitis virus G (VSV-G) and CherryPicker Red plasmids into 293 T cells. As a membrane-associated protein, CherryPicker Red was fused with “DmrA.” This domain will interact with the DmrC domain fused with Cas9 RNP to promote vesicle formation and Cas9 RNP loading. This vesicle-mediated Cas9 RNP and target gRNA can resist viral infection and inhibit viral protein expression, which provides a new solution for the effective delivery of CRISPR/Cas9 against HIV infection.

#### Engineering Exosomes Deliver Cas9 mRNA

Exosomes have been used to deliver Cas9 protein and plasmids and Cas9 mRNA. The tetraspan CD9 fusion with HuR was used to generate an RNA-binding protein domain on the inner surface of exosomes, and three AU-rich elements (AU) were added to the Cas9 mRNA sequence rich element (ARE). This high affinity for HuR and AU can efficiently load Cas9 mRNA and gRNA into CD9-HuR-modified exosomes. After intravenous administration, CD9-HuR exosomes can deliver Cas9 mRNA and gRNA to the liver to silence gene function (Li et al., 2019). The length of the Cas9 mRNA is nearly 5,000 nt, which is generally considered difficult to encapsulate into exosomes by electroporation. However, Usman et al. (2018) proved that red blood cell (RBC)-derived exosomes could be efficiently loaded with Cas9 mRNA and sgRNA by electroporation. This study achieved a gene silencing efficiency of ~32%, which implies that exosomes derived from non-nucleated cells can be used to deliver mRNA. Compared with electroporation, exosome production was greatly increased by cell nanoporation, and the mRNA transcripts enriched in the exosomes were increased by more than 1,000 times. Cells were cultured on a cell nanoperforation biochip with nanochannels to allow transient electrical pulses to pass through them and shuttle the DNA plasmid from the buffer to the attached cells. Thus, cell nanoporation technology renders exosomes universal nucleic acid carriers that require transcription operations, and this will become a useful tool to increase the exosomal loading of Cas9 mRNA (Yang et al., 2020).

In short, exosome-based nano-vehicles have opened up a new avenue for the delivery gene-editing technology and have the potential for the introduction of CRISPR/Cas9 technology into the clinic.

## Conclusion

Nanotechnology provides a toolbox for promoting CRISPR/Cas9 development. This review summarizes the most recent evidence that represents the Frontiers of Nanotechnology in gene editing. However, we are still facing many challenges when broadening the applications of this technology. In particular, the following strategies for successful clinical translation of CRISPR/Cas9 genome editing still need optimization.

After intravenous administration, the delivered CRISPR/Cas9 system needs to increase its blood circulation time and maintain its stability to protect it from degradation by extracellular nucleases. Second, the strategy should overcome the cell membrane barrier and promote cellular uptake and endosomal escape to target the cell successfully. Moreover, it is necessary to transport Cas9 into the nucleus, so that it can exert its function. Therefore, the development of safe and efficient programmable nanocarriers to overcome all these obstacles is urgently needed. Further development should focus on improving the efficiency of nanocarrier tissue and cell targeting to realize gene editing in specific tissues and cells. This goal can be fulfilled by introducing targeting ligands on nanoparticle surfaces according to different tissues or cell characteristics for targeted delivery or by designing corresponding responsive nanostructures that respond to the tissue environment.

Additionally, current nanocarriers are mainly used to knockout the corresponding genes. However, the base substitution mutation efficiency is still very low, especially for the gene editing necessary to correct some genetic diseases. Therefore, it is necessary to enhance the loading and delivery efficiency of CRISPR/Cas9 to realize *in vivo* gene editing to treat different diseases. In 2016, the first clinical trial of CRISPR/Cas9 was conducted, demonstrating the excellent prospects of nano-delivery systems for clinical gene editing. The rapid development of gene-editing technology and biomaterial science will pave the way for gene editing in clinical disease treatment in the near future.

## Author Contributions

KO, XX, LX, XZ, CW, and ZQ performed the discussion. YL drew the figures. LD, YL, and WS conceived and wrote the manuscript. All authors contributed to the article and approved the submitted version.

### Conflict of Interest

The authors declare that the research was conducted in the absence of any commercial or financial relationships that could be construed as a potential conflict of interest.
